# Out-of-pocket expenditure for hypertension care: a population-based study in low-income urban Medellin, Colombia

**DOI:** 10.1080/16549716.2020.1806527

**Published:** 2020-09-01

**Authors:** Esteban Londoño Agudelo, Anaí García Fariñas, Viviana Pérez Ospina, Cecilia Taborda Pérez, Tatiana Villacrés Landeta, Tullia Battaglioli, Rubén Gómez Arias, Patrick Van der Stuyft

**Affiliations:** a Department of Public Health, Institute of Tropical Medicine, Antwerp, Belgium; b Faculty of Medicine and Health Sciences, Department of Public Health and Primary Care, Ghent University, Ghent, Belgium; c Facultad de Medicina, Universidad CES, Medellin, Colombia; d Facultad Nacional de Salud Pública, Grupo de Epidemiología, Universidad de Antioquia, Medellín, Colombia; e Dirección de Investigación Clínica y Evaluación de Impacto, Instituto Finlay de Vacunas, La Habana, Cuba; f Dirección Ejecutiva, PSICOL -Psicología Ocupacional S.A.S, Medellín, Colombia; g UPSS Santa Cruz, Metrosalud E.S.E, Medellín, Colombia; h Facultad de Economía, Pontificia Universidad Católica del Ecuador, Quito, Ecuador

**Keywords:** Out-of-pocket expenses, chronic diseases, household budgets, catastrophic health expenditure, health insurance, primary health care, health equity, Latin America

## Abstract

Background

Hypertension requires life-long medical care, which may cause economic burden and even lead to catastrophic health expenditure.

Objective

To estimate the extent of out-of-pocket expenditure for hypertension care at a population level and its impact on households’ budgets in a low-income urban setting in Colombia.

Methods

We conducted a cross-sectional survey in Santa Cruz, a commune in the city of Medellin. In 410 randomly selected households with a hypertensive adult, we estimated annual basic household expenditure and hypertension-attributable out-of-pocket expenditure. For socioeconomic stratification, we categorised households according to basic expenditure quintiles. Catastrophic hypertension-attributable expenditure was defined as out-of-pocket expenditure above 10% of total household expenditure.

Results

The average annual basic household expenditure was US dollars at purchasing power parity (USD-PPP) $12,255.59. The average annual hypertension-attributable out-of-pocket expenditure was USD-PPP $147.75 (95% CI 120.93–174.52). It was incurred by 73.9% (95% CI 69.4%-78.1%) of patients, and consisted mainly of direct non-medical expenses (76.7%), predominantly for dietary requirements prescribed as non-pharmacological treatment and for transport to attend health care consultations. Medical out-of-pocket expenditure (23.3%) was for the most part incurred for pharmacological treatment. Hypertension-attributable out-of-pocket expenditure represented on average 1.6% (95% CI 1.3%-1.9%) of the total annual basic household expenditure. Eight households (2.0%; 95% CI 1.0%-3.8%) had catastrophic health expenditure; six of them belonged to the two lowest expenditure quintiles. Payments related to dietary requirements and transport to consultations were critical determinants of their catastrophic expenditure.

Conclusions

Out-of-pocket expenditure for hypertension care is moderate on average, but frequent, and mainly made up of direct non-medical expenses. Catastrophic health expenditure is uncommon and affects primarily households in the bottom socioeconomic quintiles. Financial protection should be strengthened by covering the costs of chronic diseases-related dietary requirements and transport to health services in the most deprived households.

Abbreviations

NCDs: Non-communicable diseases; LMICs: Low and middle-income countries; WHO: World Health Organization; HTN: hypertension; CVDs: Cardiovascular diseases; OOPE: out-of-pocket expenditure; USD-PPP: US dollars at purchasing power parity; CI: Confidence interval

## Background

Non-Communicable Diseases (NCDs) are the leading cause of death worldwide, accounting for an estimated 71% of all demises in 2016 [1]. Four out of five of these deaths occur in low and middle-income countries (LMICs) [2]. United Nations high-level meetings held in the last decade [3–5] highlighted the impact of NCDs on global health and development. Recent reports from the World Health Organization (WHO) and other international agencies also emphasised the threat that NCDs pose to developing countries and urge to scale up programs and policies to address their growing burden [6–8].

Uncontrolled hypertension (HTN) is a key modifiable risk factor for cardiovascular diseases (CVDs), the major NCDs, and is associated to more than 10 million preventable deaths each year [9]. CVDs mainly hit the middle-aged population in LMICs [10]. In Latin America and the Caribbean alone, 1.6 million people die from CVDs every year (38% of all deaths), half a million of them before 70 years of age [11]. In Colombia, 32% of deaths and 18% of Years of Life Lost in 2016 can be attributed to CVDs [12]. High HTN prevalence, poor control, and weak health systems’ response are critical factors fuelling the rising epidemic of CVDs and its increasing costs in LMICs [8,13,14].

HTN requires life-long care, but unfortunately management of NCDs is often reduced to the belated treatment of complications or acute exacerbations, in specialised settings, and at high cost [15,16]. Not surprisingly, most out-of-pocket expenditure (OOPE) for health care is related to NCDs [7,16]. Health care financing that relies on OOPE is inefficient and inequitable and creates significant access barriers [17,18]. Despite economic growth in the Americas region during the last two decades, an estimated 30% of the region’s population still has no access to care due to financial obstacles [19]. Furthermore, according to WHO and the World Bank, between 2000 and 2010 two to four million people in nine Latin American and Caribbean countries were driven into poverty by OOPE [20]. For the poor, even spending a small amount of money can take a substantial out portion of the household’s disposable income [19].

Colombian insurance schemes do not reimburse health care expenses, but directly remunerate the provider they contract with for the ambulatory care delivered as part of HTN disease management programs [21,22]. However, an exploratory service-based study [23] suggests that Colombian families often have to bear HTN-related expenses, mainly for anti-hypertensive drugs, referrals, and transport. Collecting information on OOPE can make an essential contribution to policy formulation towards universal health coverage, development of equitable health care services, financial protection and social justice [20]. Notwithstanding, population-based evidence on OOPE for HTN in the Americas derives from the high-income northern countries of the region only [24–26].

The main objective of the present study is to estimate the extent of OOPE for HTN at a population level in an urban Colombian setting and to investigate whether accessing HTN care leads to catastrophic health expenditure.

## Methods

### Study setting

The Colombian health care system has two different insurance schemes: the contributory and the subsidised. Premium payments to the contributory scheme are compulsory for formally employed workers, people receiving pensions and the self-employed with an income above the minimum monthly salary (689,455 Colombian Pesos, about 209 US dollars). The State finances the subsidised scheme. The set-up is organised along private market mechanisms, with the role of the State limited to guaranteeing competition and ordered interaction. Health insurance companies contract the provision of care with health provider institutions -private ones or autonomous public entities- based on capitation and fee-for-service payments. Regardless of scheme, affiliates and their dependents are entitled to benefits comprising a standardised health care package. Hypertensive patients are exempt from paying fees for consultations, medication and laboratory examinations at first-line health services. Cosmetic procedures, experimental treatments and health services not available in the country, amongst others, are excluded from the benefits package, but in principle – albeit not in practice- all insured have also access to secondary and tertiary care.

Our study was conducted in 2016 in the Commune of Santa Cruz, Medellin, Colombia. Medellin is the second-largest industrialised city of Colombia. In 2016, its total population was around 2.5 million [27]. Santa Cruz, one of its sixteen Communes, is located in the northeast of Medellin, and the second most deprived area of the city [28]. In 2015, it had a total population of 111,452 (53% women and 47% men) [29]. Nearly 55% of the population is insured under the contributory scheme, about 44% under the subsidised scheme and 1% does not have any insurance coverage [30]. We specifically chose this Commune because it is urban and has a predominantly low-income population, making it akin to the environments the majority of people in Latin America is currently living in. It is representative of Colombia’s national health system’s functioning, and the major health care provider in the area is committed to interventions for improving HTN control activities based on our research results.

### Sampling

We used stratified cluster sampling. To estimate the HTN prevalence with 2% precision, allowing for an alpha error of 5% and assuming 1.5 adults over 35 years per household, a prior prevalence of 18%, a design effect of 1.5 and 25% non-response, we needed to include a total of 1380 households. The municipality’s Planning Office provided the total population size of the Commune’s 11 neighbourhoods and a list of addresses. To select households, we subdivided all neighbourhoods into clusters of 15 contiguous houses and randomly selected in each neighbourhood a number of clusters proportional to its size. For the present analysis we used data on general spending and OOPE for HTN from those households that reported at least one hypertensive member aged 35 years or older.

### Data collection

Interviews were carried out during home visits by professional interviewers who had been previously trained on the administration of the study questionnaire in order to guarantee reliability and consistency. The head of the selected households responded to questions on basic spending. The first amongst the reported hypertensive family members aged 35 years or older that was encountered answered the questions on HTN-related expenses. If necessary, up to two repeated visits were made.

The questionnaire was designed to first provide general information on overall basic expenses, disaggregated by type. We enquired about food and transport costs in the two weeks leading up to the interview. Spending on utilities, housing, health care in general, clothing, and education referred to the month before the interview. In the detailed questions on OOPE for HTN we inquired about both medical and non-medical costs. Questions related to expenses for pharmaceuticals referred to the month before the interview. For spending on consultations, laboratory tests and radiology the recall period was six months. For hospitalisations and emergencies, we collected cost data for the previous year. HTN associated direct non-medical costs for items such as transport to and food bought during journeys to HTN medical appointments were recorded with the recall period used for costs on the service utilised, as was expenditure for administrative procedures. Non-pharmacological treatment costs, including additional food expenditure due to HTN dietary requirements and expenses related to increased physical activity, such as visiting a fitness centre, were explored for the week before the interview.

### Data analysis

Data were double entered in a Microsoft Excel 2010 database and analysed using the Statistical Package for Social Sciences (SPSS) V.23 (SPSS Inc., Chicago, IL, USA). We developed checks for data entry with built-in filters and logic constraints.

We estimated annual basic households’ expenditure and HTN-attributable OOPE, assuming a linear spending trend across the year. Colombian pesos were converted in US dollars adjusted at 2016 purchasing power parity (USD-PPP). For socioeconomic stratification, we categorised households according to quintiles of the annual basic expenditure in the overall study population. Catastrophic health expenditure for HTN care was defined as HTN- attributable OOPE higher than 10% of the total annual basic household expenditure [31,32], or higher than 40% of the household non-food expenditure [18].

We used mean and standard deviation, and median and quartiles, to summarise the expenditure data for descriptive purposes. Unless otherwise reported, these measures were calculated for the studied sample as a whole, i.e. including units with no expenditure on a particular item. Bootstrapping was applied to estimate 95% Confidence Intervals (CI) for the means. The Kruskal-Wallis test was used to test for differences of OOPE among socioeconomic quintiles, the Mann-Whitney test for comparisons between the contributory and the subsidised insurance schemes, and the Fisher Exact Test for differences in percentage of households with catastrophic expenditure across quintiles or between the contributory and subsidised schemes.

## Results

A total of 415 out of the 1380 sampled households had at least one member aged 35 years or older with a previous diagnosis of HTN; 67 and 8 of these families included one and two further hypertensive patients, respectively. We excluded three households with incomplete expenditure data and two with missing insurance information from the analysis. The average annual basic household expenditure was USD-PPP 12,255.59, 41.5% of which went to food (Table 1). Transport, utilities, and housing represented together 42.1% of the annual expenses, while health spending amounted to USD-PPP 757.16 or 6.2%. We found high variability and a right-skewed distribution for all expenditure components, but their means and medians were generally comparable in both insurance schemes. The mean and median total expenditure was USD-PPP 12,979.92 and 10,978.85 vs 11,535.90 and 9,296.87 for the 204 and 206 households belonging to the contributory and subsidised scheme, respectively.

**Table 1.** Annual basic household expenditure (USD-PPP) by item. Santa Cruz, Medellin-Colombia, 2016.

Item	Mean expenditure (95% CI)	Standard deviation	Median (quartiles)	% of mean total basic expenditure
Food	5,080.50 (4,559.10–5,601.97)	5,371.25	3,769.31 (2,317.78–6,502.78)	41.5
Transport	2,062.56 (1,735.20–2,389.98)	3,372.29	1,316.05 (407.24–2,477.27)	16.8
Utilities	1,607.18 (1,518.97–1,695.39)	908.54	1,545.10 (1,025.73–2,137.31)	13.1
Housing	1,495.34 (1,340.23–1,650.40)	1,597.61	822.93 (441.15–2,167.59)	12.2
Health	757.16 (674.41–839.85)	852.13	662.73 (0–1,021.47)	6.2
Clothing	743.80 (446.24–1,041.37)	3,064.83	0 (0–509.03)	6.1
Education	509.08 (345.10–673.01)	1,688.74	0 (0–322.39)	4.1
Total basic household expenditure	12,255.59 (11,387.82–13,123.29)	8,938.19	10,328.39 (6,915.11–14,928.04)	100.0

USD-PPP: US Dollars at Purchasing Power Parity; CI: Confidence Interval.

Table 2 summarises the annual basic household expenditure by item and socioeconomic quintile. The first, most deprived, to the fifth quintile contained 64.6%, 53.7%, 42.7%, 40.2% and 50.0% of households from the subsidised scheme, respectively. Expenditure on all items displayed statistically significant differences (p < 0.001) between quintiles, with a monotonous increase from the first to the fifth one. Of note, spending on health care relative to total spending was highest in the most deprived quintile (8.3%) and lowest in the wealthiest one (4.8%).

**Table 2.** Annual basic household expenditure (USD-PPP) by socioeconomic quintile.Santa Cruz, Medellin-Colombia, 2016.

Item	Socioeconomic quintile (annual basic household expenditure)				
	Less than 6,335.82	From 6,335.82 to 8,833.27	From 8,833.28 to 11,726.87	From 11,726.88 to 16,367.14	More than 16,367.14
	Mean (median) expenditure Standard deviation (quartiles)				
Food	1,904.86 (1,909.08) 1,076.63 (1,146.99–2,646.93)	2,848.34 (2,859.04) 1,395.50 (1,757.86 − 3,749.84)	4,020.75 (3,834.00) 1,954.51 (2,646.93–5,404.13)	5,883.09 (6,021.33) 2,195.01 (4,277.53–7,245.99)	10,745.59 (8,390.44) 9,186.97 (6,617.32–11,558.29)
Transport	534.66 (127.25) 803.19 (0.00–882.31)	1,263.65 (1,238.61) 1,060.19 (397.02–2,073.43)	1,927.41 (1,548.29) 1,560.40 (661.76–2,477.27)	2,028.55 (1,811.29) 1,295.77 (1,238.61–2,694.04)	4,558.60 (2,562.07) 6,496.50 (1,238.61–5,264.17)
Utilities	959.54 (1,051.81) 572.58 (610.81–1,354.89)	1,485.88 (1,511.78) 826.71 (906.75–1,968.13)	1,625.36 (1,538.28) 857.81 (1,028.22–2,219.35)	2,056.34 (1,898.10) 922.76 (1,516.92–2,667.32)	1,908.64 (1,870.89) 912.92 (1,272.57–2,453.53)
Housing	477.72 (407.24) 518.01 (152.73–551.43)	1,138.28 (661.76) 1,142.67 (441.15–1,323.46)	1,378.52 (882.31) 1,315.84 (441.15–1,879.98)	1,776.14 (1,018.06) 1,666.08 (661.76–3,054.17)	2,705.93 (2,646.93) 2,012.97 (882.31–3,826.15)
Health	362.19 (0.00) 550.51 (0.00–787.18)	470.85 (320.71) 519.58 (0.00–844.99)	677.88 (654.08) 662.14 (0.00–994.31)	1,072.47 (1,002.81) 804.81 (502.92–1,608.54)	1,202.27 (833.26) 1,207.84 (356.30–1,761.21)
Clothing	62.25 (0.00) 267.06 (0.00–0.00)	232.12 (0.00) 580.04 (0.00–0.00)	417.68 (0.00) 815.79 (0.00–509.03)	617.03 (0.00) 889.23 (0.00–1,018.06)	2,389.82 (393.67) 6,477.95 (0.00–2,036.12)
Education	43.05 (0.00) 179.01 (0.00–0.00)	102.81 (0.00) 340.56 (0.00–0.00)	205.95 (0.00) 399.73 (0.00–186.64)	579.17 (0.00) 1,055.48 (0.00–756.73)	1,614.32 (635.42) 3,355.04 (0.00–2,358.50)
Total basic household expenditure	4,344.27 (4,583.32) 1,397.29 (3,443.57–5,440.64)	7,541.98 (7,566.64) 722.87 (6,915.11–8,155.28)	10,253.60 (10,328.39) 822.06 (9,597.24–10,918.60)	14,012.80 (14,234.54) 1,309.19 (12,897.88–14,928.04)	25,125.18 (20,621.47) 11,740.07 (17,960.10–26,277.85)

USD-PPP: US Dollars at Purchasing Power Parity.

Of the 410 hypertensive patients, 383 (93.4%) were under anti-hypertensive treatment. In Tables 3 and 4 we detail their average annual OOPE for HTN care and its structure by insurance scheme and by socioeconomic quintile. 93.6% of patients in the contributory scheme but only 54.4% in the subsidised one reported HTN-attributable OOPE for at least one item. The average OOPE was USD-PPP 193.13 (95%CI 151.97–234.29) in the former against 102.76 (95%CI 69.17–136.40) in the latter scheme, in which the average expenditure on each individual item was, in general, also lower. In both schemes, payments were mainly for direct non-medical expenses, 73.6% and 82.4% of the total OOPE, respectively.

**Table 3.** Patient’s annual OOPE for HTN care (USD-PPP) by insurance scheme. Santa Cruz, Medellín-Colombia, 2016.

	Number and % of HTN patients with OOPE				Mean OOPE of HTN patients with expenditure on the item		Mean OOPE of all HTN patients		% of the total OOPE of all HTN patients	
	Contributory*		Subsidised*		Contributory	Subsidised	Contributory	Subsidised	Contributory	Subsidised
Direct medical expenses										
Pharmacotherapy	125	61.3	43	20.9	53.54	67.66	32.83	14.12	17.0	13.7
Scheduled consultations	89	43.6	9	4.4	14.98	6.65	6.54	0.27	3.4	0.3
Hospitalisations	2	1.0	4	1.9	259.60	160.36	2.54	3.14	1.3	3.1
Laboratory tests	63	30.9	10	4.9	10.92	8.17	3.35	0.38	1.7	0.4
Spontaneous consultations	11	5.4	2	1.0	41.43	6.87	2.22	0.05	1.1	0.1
Radiology	28	13.7	3	1.5	10.28	9.19	1.41	0.11	0.7	0.1
Emergency care	6	2.9	0	0.0	67.60	0.00	2.00	0.00	1.0	0.0
Sub-total medical expenses	149	73.0	49	23.8	69.71	76.20	50.89	18.12	26.4	17.6
Direct non-medical expenses										
Transport related to HTN consultations	173	84.8	73	35.4	93.78	86.80	79.50	30.77	41.2	29.9
Food bought during HTN consultations	40	19.6	25	12.1	77.56	44.46	15.20	5.41	7.9	5.3
Non-pharmacological treatment	9	4.4	12	5.8	1,039.15	830.82	45.86	48.40	23.7	47.1
Administrative procedures related to HTN consultations	2	1.0	1	0.5	169.71	20.34	1.68	0.11	0.9	0.1
Sub-total non-medical expenses	173	84.8	87	42.2	167.71	200.43	142.24	84.64	73.6	82.4
TOTAL	191	93.6	112	54.4	206.27	189.02	193.13	102.76	100.0	100.0

OOPE: out-of-pocket expenditure; HTN: hypertension/hypertensive; USD-PPP: US Dollars at Purchasing Power Parity.

*Out of 204 and 206 HTN patients insured in the contributory and subsidised scheme, respectively.

**Table 4.** Patients’ OOPE for HTN care (USD-PPP) by insurance schemes and socioeconomic quintile. Santa Cruz, Medellin-Colombia, 2016.

Item	Insurance scheme n (%) of HTN patients with OOPE	Household socioeconomic quintile (annual household expenditure)						
		All (95% CI)	Less than 6,335.82	From 6,335.82 to 8,833.27	From 8,833.28 to 11,726.87	From 11,726.88 to 16,367.14	More than 16,367.14	p-value*
		Mean OOPE per patient						
Direct medical expenses								
Pharmacotherapy	Contributory 125 (61.3)	32.80 (22.71–42.94)	22.44	29.91	31.69	51.11	22.23	0.028
	Subsidised 43 (20.9)	14.12 (8.22–20.01)	7.90	8.49	13.90	8.38	32.94	0.026
	Difference (p-value**)	18.68 (<0.001)	14.50 (<0.001)	21.42 (<0.001)	17.79 (0.056)	42.73 (<0.001)	−10.71 (0.449)	-
Scheduled consultations	Contributory 89 (43.6)	6.54 (1.51–11.57)	4.06	4.54	3.03	6.11	14.71	0.041
	Subsidised 9 (4.4)	0.27 (0.11–0.49)	0.11	0.11	1.03	0.16	0.22	0.214
	Difference (p-value)	6.27 (<0.001)	3.95 (<0.001)	4.43 (<0.001)	2.00 (0.043)	5.95 (<0.001)	14.49 (0.001)	-
Laboratory tests	Contributory 63 (30.9)	3.35 (1.95–4.81)	1.57	4.38	2.87	4.33	3.19	0.132
	Subsidised 10 (4.9)	0.38 (0.11–0.65)	0.22	0.11	0.97	0.16	0.65	0.220
	Difference (p-value)	2.97 (<0.001)	1.35 (0.001)	4.27 (<0.001)	1.90 (0.211)	4.17 (0.004)	2.54 (0.011)	-
Radiology	Contributory 28 (13.7)	1.41 (1.95–4.81)	0.49	0.54	1.68	2.97	0.76	0.734
	Subsidised 3 (1.5)	0.11 (0.11–0.56)	0.00	0.11	0.65	0.00	0.00	0.165
	Difference (p-value)	1.30 (<0.001)	0.49 (0.018)	0.43 (0.127)	1.03 (0.090)	2.97 (0.038)	0.76 (0.012)	-
Spontaneous consultations	Contributory 11 (5.4)	2.22 (0.00–5.41)	0.00	0.49	0.27	6.76	2.22	0.467
	Subsidised 2 (1.0)	0.05 (0.00–0.16)	0.00	0.00	0.38	0.00	0.00	0.044
	Difference (p-value)	2.17 (0.011)	-	0.49 (0.059)	−0.11 (0.919)	6.76 (0.095)	2.22 (0.317)	-
Hospitalisations	Contributory 2 (1.0)	2.54 (0.00–6.33)	0.00	0.00	0.00	7.19	4.06	0.631
	Subsidised 4 (1.9)	3.14 (0.00–8.81)	0.00	0.76	0.38	0.00	14.49	0.366
	Difference (p-value)	−0.60 (0.423)	-	− 0.76 (0.353)	− 0.38 (0.099)	7.19 (0.412)	−10.43 (0.986)	-
Emergency care	Contributory 6 (2.9)	2.00 (0.00–5.41)	0.59	0.43	0.00	7.57	0.00	0.205
	Subsidised 0 (0.0)	0.00 (0.00–0.00)	0.00	0.00	0.00	0.00	0.00	-
	Difference (p-value)	2.00 (0.013)	0.59 (0.054)	0.43 (0.282)	-	7.57 (0.150)	-	-
Sub-total direct medical expenses	Contributory 149 (73.0)	50.89 (35.21–66.63)	29.15	40.29	39.48	86.10	47.21	0.024
	Subsidised 49 (23.8)	18.12 (9.95–26.28)	8.22	9.57	17.36	8.65	48.30	0.004
	Difference (p-value)	32.77 (<0.001)	20.93 (<0.001)	30.72 < 0.001)	22.12 (0.003)	77.45 (<0.001)	−1.09 (0.144)	-
Direct non-medical expenses								
Transport related to HTN consultations	Contributory 173 (84.8)	79.50 (65.71–93.29)	76.31	88.97	61.22	82.26	90.59	0.551
	Subsidised 73 (35.4)	30.77 (20.77–40.72)	20.50	31.48	56.30	26.55	24.82	0.239
	Difference (p-value)	48.73 (<0.001)	55.81 (<0.001)	57.49 (<0.001)	4.92 (0.021)	55.71 (<0.001)	65.77 (<0.001)	-
Non-pharmacological treatment	Contributory 9 (4.4)	45.86 (13.47–78.20)	0.00	0.00	58.19	81.02	64.58	0.277
	Subsidised 12 (5.8)	48.40 (17.36–79.45)	26.61	55.16	18.93	16.06	120.50	0.651
	Difference (p-value)	−2.54 (0.542)	−26.61 (0.195)	−55.16 (0.103)	39.26 (0.459)	64.96 (0.321)	−55.92 (0.411)	-
Food bought during HTN consultation	Contributory 40 (19.6)	15.20 (9.46–20.93)	5.90	24.66	7.36	17.63	19.20	0.459
	Subsidised 25 (12.1)	5.41 (2.59–8.22)	3.03	4.65	8.38	4.33	7.63	0.605
	Difference (p-value)	9.79 (0.023)	2.87 (0.696)	20.01 (0.730)	−1.02 (0.307)	13.30 (0.090)	11.57 (0.336)	-
Administrative procedures related to HTN appointments	Contributory 2 (1.0)	1.68 (0.00–4.00)	7.03	0.00	0.00	0.00	3.30	0.401
	Subsidised 1 (0.5)	0.11 (0.00–0.27)	0.00	0.00	0.59	0.00	0.00	0.299
	Difference (p-value)	1.57 (0.553)	7.03 (0.176)	-	−0.59 (0.247)	-	3.30 (0.317)	-
Sub-total direct non-medical expenses	Contributory 173 (84.8)	142.24 (106.92–177.55)	89.18	113.63	126.77	180.91	177.66	0.747
	Subsidised 87 (42.2)	84.64 (51.59–117.68)	50.13	91.24	84.15	46.89	152.95	0.506
	Difference (p-value)	57.60 (<0.001)	39.05 (<0.001)	22.39 (0.001)	42.62 (0.024)	134.02 (<0.001)	24.71 (0.001)	-
Total direct medical and non-medical expenses	Contributory 191 (93.6)	193.13 (151.97–234.29)	118.39	153.92	166.25	267.01	224.88	0.124
	Subsidised 112 (54.4)	102.76 (69.17–136.40)	58.41	100.86	101.57	55.54	201.24	0.038
	Difference (p-value)	90.37 (<0.001)	59.98 (<0.001)	53.06 (<0.001)	64.68 (0.010)	211.47 (<0.001)	23.64 (0.022)	-

OOPE: out-of-pocket expenditure; HTN: hypertension; USD-PPP: US Dollars at Purchasing Power Parity; CI: Confidence Interval.

*Kruskal-Wallis test for differences across socioeconomic quintiles.

**Mann–Whitney U test for differences between Contributory and Subsidised schemes.

Overall, transport to attend health care appointments and non-pharmacological treatment -almost exclusively additional food expenditure due to HTN dietary requirements- were the critical drivers of direct non-medical expenses. Expenditure for transport was frequent, but more often than not incurred by patients in the contributory scheme only, and it entailed a significantly higher average cost in that scheme than in the subsidised one. On the other hand, only about 5% of patients in either scheme informed OOPE for non-pharmacological treatment. Notwithstanding, due to the high cost per affiliate that incurred the expenditure, it made up almost a quarter and close to half of the average overall HTN-attributable OOPE in the contributory and the subsidised scheme, respectively. Direct medical expenses, in their turn, consisted mainly of payments for pharmacotherapy and were met three times more by affiliates of the contributory scheme. Of importance, OOPE for consultations, hospitalisations and emergency care was low and infrequent in both schemes.

Regardless of expenditure item, OOPE within socioeconomic quintiles was, as a rule, significantly higher for patients from the contributory scheme (Table 4). Within each insurance scheme, the differences across the socioeconomic quintiles were usually rather minor and they rarely showed a clear trend. Nevertheless, in the contributory scheme, patients in the two better off quintiles had significantly higher expenditure for medical consultations compared to the other strata, and in the subsidised scheme OOPE for pharmacotherapy and non-pharmacological treatment tended to be mainly incurred in the fifth quintile.

We also explored the impact of OOPE for HTN on the household budget (Table 5). The attributable OOPE per hypertensive family member represented on average 1.6% of the total annual basic household expenditure. This proportion was significantly higher in the contributory than in the subsidised scheme, both overall and within each socioeconomic quintile. Furthermore, within each insurance scheme, the OOPE as percentage of basic expenditure was different between quintiles and, for both schemes confounded, significantly higher for the most deprived ones, attaining 2.3% in the bottom quintile compared to 1.1% in the fifth quintile. OOPE for HTN as percentage of the households’ non-food expenditure showed the same pattern. Overall, it attained 2.9%, with a gradient over quintiles and higher percentages in the contributory scheme.

**Table 5.** Summary indicators of OOPE for HTN care. Santa Cruz, Medellin-Colombia, 2016.

Summary indicator	Insurance scheme		Socioeconomic quintile (annual household expenditure, USD-PPP)					
		All households	Less than 6,335.82	From 6,335.82 to 8,833.27	From 8,833.28 to 11,726.87	From 11,726.88 to 16,367.14	More than 16,367.14	p- value (* or ***)
OOPE per HTN patient as % of household expenditure								
OOPE for HTN care as % of basic household expenditure	All	1.55	2.27	1.69	1.40	1.31	1.08	*0.287*
	Contributory	1.83	2.53	2.01	1.70	1.93	1.18	*<0.001*
	Subsidised	1.27	2.12	1.40	0.99	0.38	0.98	*0.289*
	p-value**	*<0.001*	*<0.001*	*<0.001*	*0.008*	*<0.001*	*0.032*	-
OOPE for HTN care as % of non-food household expenditure	All	2.88	3.45	3.26	2.95	2.66	2.02	*0.398*
	Contributory	3.33	3.96	3.29	3.35	3.98	2.14	*0.001*
	Subsidised	2.40	3.17	3.23	2.41	0.69	1.91	*0.323*
	p-value**	*<0.001*	*<0.001*	*0.001*	*0.004*	*<0.001*	*0.011*	-
Number (%) of households with catastrophic expenditure due to OOPE for one HTN patient								
OOPE for HTN care over 10% of basic household expenditure	All	8 (2.0)	4 (4.9)	2 (2.4)	2 (2.4)	0 (0.0)	0 (0.0)	0.091
	Contributory	2 (1.0)	0 (0.0)	0 (0.0)	2 (4.3)	0 (0.0)	0 (0.0)	0.145
	Subsidised	6 (2.9)	4 (7.5)	2 (4.5)	0 (0.0)	0 (0.0)	0 (0.0)	0.118
	p-value***	0.284	0.292	0.497	0.505	-	-	
OOPE for HTN care over 40% of non-food household expenditure	All	2 (0.5)	1 (1.2)	1 (1.2)	0 (0.0)	0 (0.0)	0 (0.0)	0.198
	Contributory	0 (0.0)	0 (0.0)	0 (0.0)	0 (0.0)	0 (0.0)	0 (0.0)	-
	Subsidised	2 (1.0)	1 (1.9)	1 (2.3)	0 (0.0)	0 (0.0)	0 (0.0)	0.741
	p-value***	0.499	1.000	1.000	-	-	-	

OOPE: out-of-pocket expenditure; HTN: hypertension/hypertensive; USD-PPP: US Dollars at Purchasing Power Parity.

* Kruskal-Wallis Test for differences in OOPE for HTN across socioeconomic quintiles.

**Mann–Whitney U test for differences in OOPE for HTN between contributory and subsidised schemes.

*** Fisher Exact Test for differences in % of households with catastrophic expenditure between contributory and subsidised schemes or across quintile.

Eight households in total (2.0%; 95% CI 1.0%-3.8%) had OOPE for one hypertensive family member surpassing 10% of their annual basic expenditure, of which six belonged to the two bottom quintiles. At the above threshold, catastrophic spending for HTN was more frequent, but not significantly so, among households enlisted in the subsidised scheme. The four households with catastrophic expenditure that belonged to the bottom socioeconomic quintile had, for the family as a whole, a basic expenditure below 60% of one minimum salary. The main drivers of catastrophic expenditure for the in total six families from the subsidised scheme were food prescribed as dietary requirement for non-pharmacological treatment (4), transport for HTN consultations (2) and anti-hypertensive drugs (1). For the two households affiliated to the contributory scheme, catastrophic OOPE was mainly due to expenditure for both non-pharmacological treatment and anti-hypertensive medication.

## Discussion

OOPE for HTN care was reported by 93.6% and 54.4% of the hypertensive patients and amounted, on average, to USD-PPP 193.13 and 102.76 per year for the insured in the contributory and subsidised schemes, respectively. Non-medical OOPE represented 73.6% of the total OOPE in the contributory and 82.4% in the subsidised scheme and was mainly made up by expenses for transport to HTN appointments and for non-pharmacological treatment, essentially food related to HTN dietary requirements. The former cost was particularly frequent in the contributory scheme, the latter infrequent in both schemes. Medical OOPE was higher on average in the contributory scheme and experienced some three times more frequently than in the subsidised one. It was, in both schemes, mostly brought on by pharmacological treatment, while remarkably low for consultations, hospitalisations and emergency care. HTN-attributable OOPE for a hypertensive patient represented on average 1.6% of the total annual basic household expenses and 2.9% of the non-food expenditure. These proportions were higher for the bottom socioeconomic quintiles and within quintiles higher in the contributory scheme. In two percent of households it led to catastrophic health expenditure due to the HTN care. The risk was higher, but not significantly so, among households in the subsidised scheme and overall three quarters of the affected belonged to the most deprived quintiles. Financial costs related to HTN dietary requirements stood out as the critical determinant, followed by payments for anti-hypertensive medication and transport for HTN consultations.

Our study’s main limitation is having been conducted in a low-income urban setting. Almost half of the Colombian population lives in comparable environments, but our results may not reflect the situation in upper-class urban zones or in underserved rural areas. The use of a questionnaire for measuring expenditure also entails potential drawbacks, related to respondents’ willingness to disclose financial information and ability to correctly recall actual expenses. The latter was mitigated by choosing variable recall periods, adapted to the nature of the different examined expenditure items. Finally, selecting just one household member with HTN to collect data on OOPE on, will have resulted in somewhat underrating the proportion of families in which this condition leads to catastrophic expenditure. However, our estimates of OOPE in itself and of its effect on household budgets for one hypertensive patient are unbiased.

Making use of information on household expenditure instead of income is an asset to our study. It better reflects the longer-term financial state of affairs, particularly in resource-constrained settings [33–36]. The study’s major strength is providing yet unavailable population-based evidence on the magnitude of OOPE for HTN care in Colombia, on its impact on household budgets and on HTN-attributable catastrophic health expenditure. Our findings could guide policymakers in strengthening financial protection and ensuring equitable access to care for families belonging to deprived socioeconomic population segments.

The basic household expenses we document are compatible with Santa Cruz Commune’s household income reported by Medellin’s government [37]. Moreover, food expenditure making up the highest share for the lowest quintile, followed by housing and transport, is in line with the patterns observed in Cauca, Colombia [38] and with regional findings [39]. This confers face value to our data. Also, it is a finding of note that over 40% of the households belonging to the first and second socioeconomic quintiles are insured under the contributory scheme, but it is not surprising given the Commune’s low-income profile and many of its formally employed, which are therefore affiliated to that scheme, earning meagre salaries.

The -in absolute terms- fairly low OOPE for HTN found in this study is in line with Colombia’s overall OOPE for health that represents a moderate 16% of the country’s total health expenditure [19]. Nevertheless, low OOPE is not always an indication of equitable access since it may be due to lack of utilization of health services [20]. In Colombia, despite 96% health insurance coverage [40], almost a third of the population reports financial barriers to access care [19]. A 2015 report further indicates that the households in the lowest socioeconomic quintile had the highest OOPE for health [41], which is in line with the findings of a literature review on the global situation in LMICs [42].

In the present study the magnitude of OOPE per patient for HTN was lower in the bottom socioeconomic quintile, where it accounted for 2.3% of the total basic household spending, than in the other ones. It led to catastrophic expenditure in 4.9% of households in the former quintile, in contrast to 1.2% in the better off segment of the population. Both figures may seem negligible in comparison with a staggering 23% catastrophic expenditure attributable to HTN in households with a hypertensive member in the rural areas of Shaanxi Province, China [43], but WHO sets the threshold for good performance with regard to financial protection in the Latin America and the Caribbean region at 2% catastrophic overall health expenditure [19]. Precisely equal to the – from that perspective quite alarming- 2.0% found in our study for just one particular disorder in one household member. Furthermore, HTN is a very prevalent condition in Colombia [12] and a common reason for outpatient care that has been exempted from user fees and co-payments.

The health system has succeeded in reducing to a minimum the OOPE for consultations, hospitalisations and emergency care for hypertensive patients and seems to protect from catastrophic expenditure due to major health shocks. However, particularly for patients enrolled in the contributory scheme, transport expenses related to attending HTN care appointments and, to a lesser extent, payments for pharmaceuticals generate non negligible OOPE. The former reveals the fragmentation of the Colombian health system and its disintegrated care provision. Health insurance companies of the contributory scheme tend to vertically integrate their facilities following profit maximising efficiency criteria rather than accommodating for the territorial distribution of users.

While around half of the more than 110,000 Santa Cruz dwellers are enlisted in the contributory scheme, they avail of no facilities providing health services in the Commune. Moreover, contributory insurance companies do not contract the provision of primary health care to the public health facilities operating in the area. In order to be attended, be it just for blood pressure control, contributory scheme affiliates are forced to travel downtown or to the South/South-west communes of the city, where most of the scheme’s health care facilities are located. Besides creating geographical barriers to care, it substantially contributes to patients insured in this scheme having overall higher OOPE for HTN than to those enlisted in the subsidised one. The OOPE for anti-hypertensive medication in the contributory scheme also finds its main origin in the geographically remote location of the insurers’ care facilities and the pharmacies linked to them [44]. It gives rise to patients acquiring drugs in nearby home private outlets – a problem compounded, in both schemes, by occasional stock-outs. It has been argued that insurer-based vertical integration does not generate distortions in the larger Colombian cities [45], but our findings suggest that ‘a market-driven vertical integration between health care insurers and providers, based on cost-containment mechanisms’ [46], is affecting not only the provision of care but also access and financial protection.

A final finding of importance is that in spite of a small proportion of patients incurring expenses related to HTN dietary requirements, the amount spent by the ones that do so is high on average and a main driver of catastrophic health expenditure, in particular in the lowest socioeconomic quintile. This is in line with evidence that healthier dietary patterns entail cost about 1.50 USD per person a day higher than the least healthy ones [47] and that food insecurity primarily affects people living on stagnant wages in poor urban communities [48,49]. Our findings highlight the vulnerability of the most deprived urban households, for whom even modest expenses to comply with medical dietary prescriptions can lead to financial hardship.

## Conclusions

OOPE for HTN care is moderate but frequent in this Colombian low-income urban community and for the larger part made up of direct non-medical expenses. Catastrophic health expenditure for HTN is infrequent, but affects some 5.0% of households in the bottom socioeconomic quintile. Our findings point to the financial vulnerability of these households. They also highlight the fragmented and disintegrated nature of the Colombian health care system. While this suggests a need for more fundamental structural reforms we can, strictly based on our results, recommend implementing a public policy measure aimed at strengthening financial protection: cover, for the most deprived households, the costs of chronic disease related dietary requirements and of transport for attending health services. Furthermore, decentralising the contributory insurance scheme’s primary care facilities should reduce, in itself, overall out-of-pocket expenditure in affiliated members.

## Paper context

The paper provides novel population-based evidence on the magnitude and structure of out-of-pocket expenditure for hypertension care and hypertension-attributable catastrophic health expenditure in low-income urban Colombia. Our findings indicate moderate direct medical expenses, but reveal the need for reimbursement mechanisms that financially protect the socioeconomically most deprived households from chronic diseases-related catastrophic direct non-medical health expenditure. Decentralising the contributory insurance scheme’s primary care facilities should reduce overall out-of-pocket expenditure in affiliated members.
